# Comparison of the clinical effectiveness of US grading scoring system vs MRI in the diagnosis of early rheumatoid arthritis (RA)

**DOI:** 10.1186/s13018-017-0653-5

**Published:** 2017-10-17

**Authors:** Huajun Xu, Yingchun Zhang, Huimei Zhang, Caishan Wang, Pan Mao

**Affiliations:** 1 Department of Ultrasound, The Second Affiliated Hospital of Soochow University, San Xiang Road 1055, Suzhou, 215004 China; 2 Department of Ultrasound, Huzhou Central Hospital, Hong Qi Road 198, Huzhou, 313000 China; 3 Department of Radiology, Huzhou Central Hospital, Hong Qi Road 198, Huzhou, 313000 China

**Keywords:** Ultrasound, Early rheumatoid arthritis, MRI, DAS28 score

## Abstract

**Background:**

As an irreversible disease, a treatment delay can negatively affect treatment response in rheumatoid arthritis (RA). Ultrasound and MRI have played an important role in assessing disease progression and response to treatment in RA for many years. The present study was designed to compare the diagnostic efficacy of ultrasound grading and MRI in early RA.

**Methods:**

In this retrospective study, 62 early RA patients within 12 months of symptom onset were included. DAS28, rheumatoid factor (RF), CRP, ESR, and anti-cyclic citrullinated peptide antibody (CCP) of the patients were measured. Bilateral hand joints and wrists were examined by ultrasonography (US) and MRI; diagnosis outcome was compared. Relationship between DAS28 scores, laboratory parameters, and ultrasound findings were analyzed.

**Results:**

Ultrasound and MRI had an equivalent diagnosis value in synovitis, joint effusion, and tenosynovitis. The detection rate of synovitis, arthroedema, and tenosynovitis on ultrasound and MRI was very close (*P* > 0.05). The detection rate of bone erosion was lower in ultrasonography than that in MRI (*P* < 0.05). There were significant differences between power Doppler ultrasonography (PDUS) and gray-scale ultrasonography (GSUS) in the diagnosis of synovitis (*χ* ^2^ = 3.92, *P* < 0.05); the sensitivity of GSUS was better than that of PDUS (*P* < 0.05). PDUS was positively correlated with DAS28, ESR, CRP, and CCP (*P* < 0.01), but not correlated with RF and disease duration (*P* > 0.05). GSUS was positively correlated with RF and CRP (*P* < 0.01), but not correlated with DAS28, CCP, ESR, and disease duration (*P* > 0.05). Bone erosion was positively correlated with disease duration, CCP, and RF (*P* < 0.01) and was not correlated with DAS28, ESR, and CRP (*P* > 0.05).

**Conclusion:**

Ultrasonography has a high reliability in the diagnosis of early RA in synovitis, joint effusion, tenosynovitis, and bone erosion. Ultrasonography and clinical and laboratory parameters had a great correlativity. Both ultrasound and MRI are effective techniques. In view of the advantages of low cost and convenience, ultrasound may be a better choice during early RA diagnosis.

## Background

Rheumatoid arthritis (RA) is a chronic, systemic inflammatory disorder that can inflict joint destruction and malformation resulting in functional disability [1, 2]. A delay in initiating therapy could adversely affect treatment outcomes such as disease activity, remission, functional capacity, and radiographic progression [3–5]. The pathophysiology of RA is not completely understood, and no single test or gold standard exists to confirm the diagnosis. Hence, the diagnosis is made based on a set of findings and symptoms typical for the RA phenotype rather than measurement of the specific pathogenic processes that lead to this phenotype [3]. Early RA is most likely to erode wrist, metacarpophalangeal, and interphalangeal joints [6, 7]; synovial pannus may cause gradual erosion of the articular cartilage and bone cortex after its formation, so early diagnosis and effective treatment is very important [8, 9].

Conventional radiography remains the mainstay for evaluation of RA patients in daily practice [10, 11]. However, as the X-ray shows late signs of disease activity and destruction of cartilage or bone, other medical imaging techniques such as ultrasonography (US) and MRI are used in RA in order to assess the earlier signs [12]. Musculoskeletal ultrasound is a readily available, useful, and versatile imaging modality with high patient acceptability [13]. In patients with arthritis, gray-scale ultrasonography (GSUS) is more sensitive than clinical examination for detecting synovitis [14, 15] and more sensitive than conventional radiography for detecting bone erosions [15, 16]. Power Doppler (PD) has been introduced for the assessment of synovitis and may provide additional information [17, 18]. Musculoskeletal ultrasound has been confirmed to be more accurate than clinical inspection in detecting synovitis and tenosynovitis [19]. The initiation of synovial inflammation is characterized by periarticular vasodilatation followed by synovial proliferation, which is accompanied by angiogenesis resulting in intra-articular new blood vessel formation. Power Doppler US (PDUS) makes it possible to discriminate between peri- and intra-articular blood flow in microvessels and to demonstrate synovial proliferation [20], while GSUS mainly assess the abnormalities of synovial morphology caused by synovitis [21].

Previously, some simplified ultrasound scoring methods have been reported and analyzed correlatively with clinical manifestations [22]. But there is a lack of contrast between clinical, laboratory, and radiologic imaging. The sensitivity and specificity of ultrasound inflammatory parameters (GSUS and PDUS) for the diagnosis of synovitis are not yet clear [23]. Magnetic resonance imaging (MRI) can directly visualize the bone and soft tissues in three dimensions and has the potential to measure inflammatory activity and joint destruction [24]. The sensitivity of ultrasound for detecting joint inflammation relative to MRI is yet to be determined.

The primary objective of this study was to investigate the sensitivity and specificity of ultrasonography (GSUS and PDUS) compared to MRI in early RA diagnosis and to compare the detection rate between ultrasound and MRI in terms of synovitis, joint effusion, tenosynovitis, and bone erosion. The secondary objective was to analyze the correlation between laboratory parameters and ultrasound findings and to analyze the reliability of each parameter of ultrasound in early RA diagnosis.

The present study was designed to compare the diagnostic efficacy of ultrasound grading and MRI in early RA. In this study, the wrist, metacarpophalangeal, and proximal interphalangeal joints were examined by ultrasound grading; the ultrasonographic features of the lesions were observed and compared with MRI and clinical and laboratory parameters; relationship between DAS28 scores, laboratory parameters, and ultrasound findings were analyzed.

## Methods

### Patients

From January 2012 to June 2016, 62 early RA patients in the outpatient department and inpatient department of Rheumatology in our hospital were enrolled in our study. This study was approved by the ethics committee of the local hospital, and informed consent was obtained from all patients. All patients underwent routine medical history inquiry, physical examination, and laboratory examination such as ESR, CRP rheumatoid factor (RF), and anti-cyclic citrullinated peptide antibody (CCP). In these 62 patients, 1364 joints of the wrist, metacarpophalangeal, and proximal interphalangeal were both examined by color Doppler ultrasonography and MRI.

Inclusion criteria include the following: within 12 months of RA symptom onset and diagnosis of RA was based on 2010 ACR/EULAR Early RA Classification and Scoring Criteria [25]. The selection of the early RA patients was supervised by two experienced rheumatologists.

The exclusion criteria were as follows: age < 18; history of rheumatoid arthritis > 2 years; been treated with anti-rheumatic drugs (methotrexate, chloroquine, leflunomide, NSAIDs, and salazosulfadimidine) previously; history of glucocorticoid usage in the past 3 months; and history of joint trauma, bacterial infection (such as purulent arthritis), or surgery.

### Clinical data collection

General clinical data including gender, age, course of disease, and laboratory parameters such as ESR, CRP, RF, and CCP were collected. Clinical physical examination was conducted by the same doctor attending the Department of Rheumatism, to simplify the examination of 28 joints. TJC28 and SJC28 were recorded and DAS28 was calculated.

DAS28 was calculated as follows. (1) TJC28 (tender joint count): a total of 28 cases of bilateral metacarpophalangeal joint, proximal interphalangeal joint, wrist joint, elbow joint, shoulder joint, and knee joint were examined and TJC28 was calculated; (2) SJC28 (swollen joint count): check the swelling situation of the above 28 joints and calculate the SJC28. DAS28 = [0.56 × SQRT (TJC28) + 0.28 × SQRT (SJC28) + 0.70 × Ln (ESR)] × 1.08 + 0.16. DAS value > 5.1 indicates high disease activity; DAS < 3.2 indicates low disease activity; and DAS < 2.6 indicates disease remission [26].

### Ultrasonography

MyLab70 (Biosound Easote), PHILIPS iU 22 high-grade color ultrasonic diagnostic apparatus, and a 10~18-MHz linear array probe were used for ultrasonic inspection. Low-pass filter, pulse repetition frequency (1000~1800 Hz), and no Doppler signal which appear behind the bone cortex are regarded as the advisable maximum gain. The inspection was performed by two ultrasound doctors who had more than 5 years of experience with musculoskeletal ultrasonography, and the two doctors had similar musculoskeletal experience. The transducer was placed in the wrist region, metacarpophalangeal (MCP), and proximal interphalangeal (PIP) joints on the dorsal and palmar view, bilaterally. All the joints were assessed in transversal and longitudinal scans. A total of 1364 joints of the wrist, metacarpophalangeal, and proximal interphalangeal joints of the 62 patients were examined. Inflammatory changes and joint structural damage of ultrasound were recorded; GSUS synovial hyperplasia, PDUS color signal, and bone erosion were graded with a semi-quantitative method. A higher score between the scores of metacarpophalangeal and proximal interphalangeal joints was taken as a representative. The ultrasound images were assessed by the above ultrasound doctors. The interrater reliability of the two ultrasonographers during the obtainment of the GSUS and PDUS was evaluated by *κ* statistics (*κ* = 0.75–0.85). Once divergence occurred during the ultrasound grading evaluation, the two parties shall solve the difference through consultation.

The following are the US classification standards [22]:

1) Synovial thickening (GSUS)

Synovial thickening was analyzed as follows: grade 0 (absence), grade 1 (small hypoechoic/anechoic line beneath joint capsule), grade 2 (joint capsule elevated parallel to joint area), and grade 3 (strong distension of joint capsule) [27, 28].

2) PDUS

PDUS was performed for synovitis and tenosynovitis in each scanning plane described above. The semi-quantitative findings of PDUS activity for synovitis were scored as follows: grade 0 = no intra-articular color signal; grade 1 = up to three single color signals or two single color signals and one confluent color signal representing only low flow; grade 2 = < 50% of the intra-articular area filled with color signals representing clear flow; and grade 3 = 50% of the intra-articular area filled with color signals.

3) Bone erosion score

The following are the bone erosion scores: grade 0 = continuous cortical bone; grade 1 = the surface of the bone cortex was not smooth, but there was no obvious bone defect in two perpendicular sections; grade 2 = cortical bone defects can be seen in two perpendicular sections; and grade 3 = extensive bone defects on the surface of the cortical bone.

4) Tenosynovitis

The sonogram showed a thickening of the tendon, reduced and uneven echo, unclear normal fibrous structure, irregular margin, and edema in the surrounding tissues. Blood flow signal in the tendon sheath can be detected by power Doppler US (PDUS) [29]. Ultrasound showed normal tendon sheaths were recorded as negative (0 points) and the abnormality was recorded as positive (1 point).

5) Joint effusion

The ultrasound of the joint effusion showed no echo or hypoechoic area in the articular cavity, to be compressible, and no color Doppler flow signal. The thickness of articular cavity effusion < 2 mm was recorded as negative (0 points) and > 2 mm was recorded as positive (1 point).

### MRI examination

GE Signa HDX 3.0 T MRI scanner (GE, USA) was used for MRI examination. All 62 patients were placed in a prone position with hands flat over the head and placed in the wrist joint coil; the hand back was fixed with a tape, so that the metacarpal and phalanx were placed in a same plane. MRI scanning sequences included SE T1WI coronal plane (TR 300 ms, TE 14 ms, matrix 512 × 256, layer thickness 3 mm, interval 0.5 mm), FastSE (FSE) T2WI coronal plane (TR 2000 ms, TE 42 ms, matrix 384 × 224, layer thickness 4 mm, interval 0.5 mm), and axial plane. Sixty-two patients underwent bilateral wrist MRI, and the MRI tablets were diagnosed by two Deputy Chief MRI diagnostic physicians. Once divergence occurred during MRI examination, the two parties shall solve the difference through consultation.

### Statistical analysis

Quantitative data for normal distribution were expressed as means ± standard (SD); the skew distribution data were expressed as median (M) and quartile spacing (Q); qualitative data was expressed as rate. SPSS 22.0 (SPSS Inc., USA) was used for statistical analysis. The detection rates of synovitis, tenosynovitis, joint effusion, and bone erosion were compared by paired chi-square test. The differences in the assessment of synovial fluid between GSUS and PDUS were compared using the paired chi-square test. Spearman rank correlation analysis was used to evaluate the correlation between the indexes of ultrasonic grading and clinical and laboratory parameters. *P* < 0.05 indicated that the difference was statistically significant.

## Results

### General clinical data of all patients

Sixty-two early RA patients (13 males/49 females) were included in our experiment. The general clinical data of all patients and the ultrasonic classification index are listed in Tables 1 and 2, respectively.

**Table 1:** General clinical data of all patients

Item	
Gender (male/female)	13/49
Mean age (years)	42.5 ± 12.1
Mean duration of disease (months)	7.6 ± 3.5
DAS28 score	4.14 ± 1.24
CRP (mg/L)	31.12 ± 11.25
ESR (mm/h)	42 ± 12.05
RF (*n*/%)	38/61.29%
CCP (+) (*n*/%)	33/53.23%
Tenosynovitis (*n*/%)	19/31.23%
Joint effusion (*n*/%)	18/29.55%
GSUS score (M/Q)	2.0/1.75
PDUS score (M/Q)	1.0/0.75
Bone erosion score (M/Q)	1.0/1

Data presented as means ± SD, or *n* patients

*ESR* erythrocyte sedimentation rate, *CRP* C-reactive protein, *CPP* anti-cyclic citrullinated peptide antibody, *GSUS* gray-scale ultrasonography, *PDUS* power Doppler ultrasonography

**Table 2:** Ultrasonic classification index (joint number: *n* = 1364)

Ultrasonic indicators	Points	Constituent ratio
GSUS score	0	290 (21.26%)
	1	285 (20.89%)
	2	708 (51.91%)
	3	81 (5.94%)
PDUS score	0	352 (25.81%)
	1	652 (47.80%)
	2	283 (20.75%)
	3	77 (5.65%)
Bone erosion score	0	792 (58.06%)
	1	306 (22.43%)
	2	218 (15.98%)
	3	48 (3.52%)
Tenosynovitis	0	938 (68.77%)
	1	426 (31.23%)
Joint effusion	0	961 (70.45%)
	1	403 (29.55%)

*GSUS* gray-scale ultrasonography, *PDUS* power Doppler ultrasonography

### Comparison of ultrasonography and MRI examination in the diagnosis of RA

When the PDUS or GSUS score is ≥ 1, the diagnostic result is considered positive for early RA.

The detection rates of synovitis, tenosynovitis, arthroedema, and bone erosion were compared. The detection rates of synovitis, arthroedema, and tenosynovitis on ultrasound and MRI were very close (*P* > 0.05). The detection rate of bone erosion was lower in ultrasonography than in MRI (*P* < 0.05) (Table 3).

**Table 3:** Comparison of ultrasonography and MRI examination in the diagnosis of RA (joint number: *n* = 1364)

Method	Synovitis	Joint effusion	Tenosynovitis	Bone erosion
US	1074 (78.74%)	403 (29.55%)	426 (31.23%)	572 (41.94%)
MRI	1053 (77.20%)	420 (30.79%)	446 (32.70%)	886 (64.96%)
*χ* ^2^	0.94	0.50	0.67	145.26
*P*	0.33	0.48	0.41	0.0001

When the PDUS or GSUS score is ≥ 1, the diagnostic result is considered positive for early RA

### Analysis of the evaluation of synovitis by ultrasonography

**Fig. 1:**
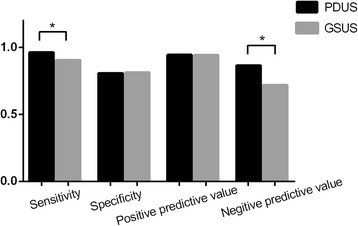
The sensitivity, specificity, positive predictive value, and negative predictive value of GSUS and PDUS

As is shown in Table 4 and Fig. 1, there were 1074 GSUS-positive and 1012 PDUS-positive joints among all 1364 joints, with a positive rate of 78.74 and 74.20%, respectively. There were significant differences between PDUS and GSUS in the diagnosis of synovitis (*χ* ^2^ = 3.92, *P* < 0.05); the sensitivity of GSUS was better than PDUS (*P* < 0.05).

**Table 4:** Comparison of GSUS and PDUS in the evaluation of synovitis

Ultrasonic indicators	Positive/negative	Positive rate	*χ* ^2^	*P*
GSUS	1074/290	78.74%	7.83	< 0.05
PDUS	1012/352	74.20%

### Correlation between ultrasonography and clinical and laboratory parameters

According to the results of Spearman rank correlation analysis, PDUS was significantly positively correlated with DAS28, ESR, CRP, and CCP (*P* < 0.01), while no significant correlation was found between PDUS, RF, and course of disease. GSUS was positively correlated with RF and CRP (*P <* 0.01), and there was no significant correlation with DAS28, CCP, ESR, and course of disease. Significant positive correlation was found between bone erosion and duration of disease, CCP, and RF (*P <* 0.01), and there was no significant correlation with DAS28, ESR, and CRP (*P* > 0.05) (Table 5).

**Table 5:** Correlation between the indexes of ultrasonic grading and clinical and laboratory parameters

Clinical and laboratory parameters	*r* (GSUS)	*r* (PDUS)	*r* (bone erosion)
Duration of disease	0.09	0.16	0.40*
DAS28	0.13	0.39*	− 0.15
CRP	0.31*	0.39*	0.16
ESR	0.13	0.41*	− 0.05
RF	0.30*	0.12	0.35*
CCP	0.18	0.29*	0.37*

*P < 0.05, statistically significant

## Discussion

With the development of high-frequency ultrasound technology, ultrasound plays an increasingly important role in the early radiographic imaging of RA. The thickened synovial tissue can be observed by GSUS, and the low velocity blood flow signal in synovial tissue can be displayed by PDUS, which is of great significance for clinical diagnosis and treatment of RA [30]. Previously, some simplified ultrasound scoring methods have been reported and analyzed correlatively with clinical manifestations. Luz et al. proposed a novel ultrasound scoring system for hand and wrist joints (US10) and for evaluation of patients with early RA and correlated the US10 with clinical, laboratory, and functional variables. The proposed US10 scoring system proved to be a useful tool for monitoring inflammation and joint damage in early RA [31].

**Fig. 2:**
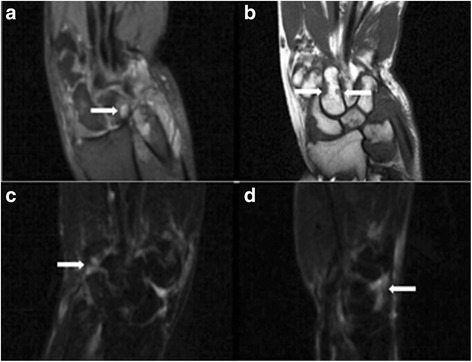
Typical case: 52-year-old female diagnosed with RA for 1 year. MRI: carpal synovitis with bone destruction. **a** T2WI: oval high signal within the lunare bone—pannus formation. **b** T1WI: carpal bones showed low signal loss—bone destruction. **c**, **d** T2WI: carpal synovitis

**Fig. 3:**
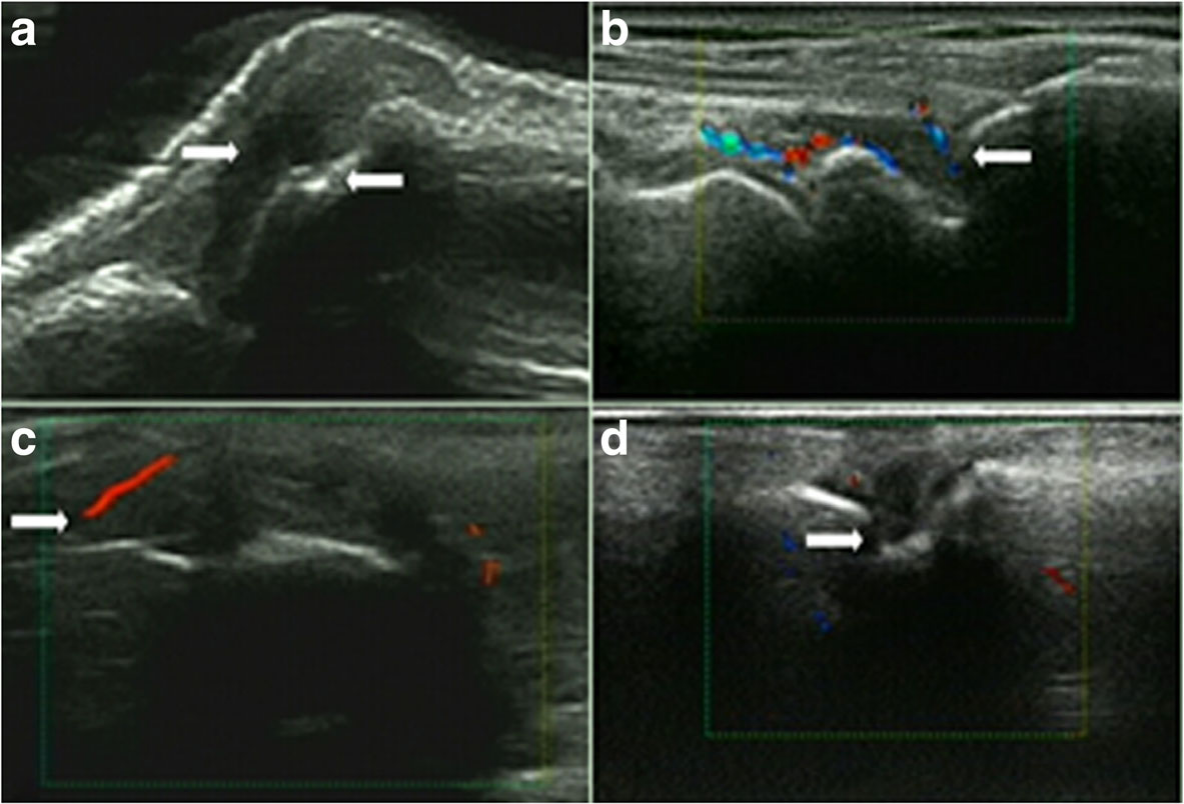
Typical case: 52-year-old female diagnosed with RA for 1 year. US: synovial hyperplasia, synovitis with bone destruction. **a** Synovial hyperplasia (2 points) and bone erosion (1 point) of the radiocarpal joint. **b** Synovitis of the scapholunate joint (PDUS: 1 point). **c**, **d** Synovial hyperplasia (GSUS: 2 points), synovitis (PDUS: 1 point), and bone erosion (2 points) of the scapholunate joint

As the joint capsule, synovial membrane, tendons, ligaments, and other soft tissue attached to the bone surface, in the relatively simple anatomy of the limb joints, these soft tissues are easy to be scanned by ultrasound. At present, ultrasound has a high reliability in the diagnosis of inflammatory lesions of RA. The application value of ultrasound diagnosis has been widely recognized by rheumatologists [32–34]. In the evaluation of joint structure, our study showed that 572 cases of bone destruction were detected by ultrasound, while 886 cases were detected by MRI (Figs. 2 and 3). The comparison of GSUS and PDUS in synovitis evaluation showed that GSUS was superior to PDUS in diagnostic sensitivity and negative predictive values; the diagnostic specificity and positive predictive value were not significantly different between GSUS and PDUS. The results of GSUS and PDUS confirmed the synovial tissue congestion and inflammatory thickening changes of early RA. Studies have shown that this subclinical synovitis is closely related to the structural damage of RA patients [35, 36]. Effective treatment can eliminate the blood flow signal in PDUS, which has a positive effect on prolonging the remission of disease in RA patients.

The presentation of ultrasound on synovial inflammation was related to RA disease activity; PDUS can better reflect the disease activity [37]. No significant correlation was found between GSUS and DAS28, CCP, ESR, and course of disease (*P* > 0.05); this may be related to the slow thickening of synovial membrane and regression of inflammation during early RA. Significant positive correlation was found between bone erosion and duration of disease, CCP, and RF; this suggested that bone erosion is a progressive destructive process in rheumatoid arthritis and it is irreversible once it appears. Traditional X-ray lacks sensitivity to early bone erosion [38, 39].

Although it has been proven that MRI has a strong correlation with histological data and provides a predictive value in structural joint damage, MRI is rather expensive, time-consuming, not always available for routine examinations, and difficult to reproduce [40, 41]. US, by its increased degree of resolution due to high-frequency transducers, constitutes a reliable and compulsory method to diagnose and monitor RA patients. Unlike MRI, US is relatively cheap, is available, and can be used as many times as necessary during patient examination, improving the exactitude of clinical examination [42–44]. Ultrasound and MRI have similar effects on the diagnosis of characteristic RA lesions [45]. Because of the advantages of economy, convenience, no radiation, good repeatability, and so on, ultrasound has been widely used in the limb joints. The value of ultrasound in early RA diagnosis and disease surveillance was highly emphasized in the guidelines for the early diagnosis of RA in 2013 [46, 47].

There are some limitations in this study: (1) the group was limited in the number of patients; a larger group of patients would probably have strengthened the results. (2) Due to its physical properties, acoustic waves cannot effectively penetrate the cortex, so ultrasound cannot assess the true situation of bone marrow edema. (3) Unlike X-ray, CT, and MRI, ultrasound cannot provide complete information about the structure of the joint due to its spatial resolution; thus, the reliability of ultrasonic diagnostic information is subject to the doctor’s operating experience to a certain extent. (4) The assessment of a single selected US image instead of a real-time examination of the joints performed by the second rheumatologist obviously introduces bias into the study. However, this is the standard way to record US examination in daily practice, and the images for a second reading were chosen by an experienced sonographer.

## Conclusion

Ultrasonography has a high reliability in the diagnosis of early RA in synovitis, joint effusion, tenosynovitis, and bone erosion. There was a good correlation between ultrasonography and clinical and laboratory parameters. Both ultrasound and MRI are effective techniques. In view of the advantages of low cost and convenience, ultrasound may be a better choice during early RA diagnosis.

## Abbreviations

CCP: Anti-cyclic citrullinated peptide antibody
GSUS: Gray-scale ultrasonography
PD: Power Doppler
RA: Rheumatoid arthritis
RF: Rheumatoid factor
